# Tetraspanin 1 as a mediator of fibrosis inhibits EMT process and Smad2/3 and beta‐catenin pathway in human pulmonary fibrosis

**DOI:** 10.1111/jcmm.14258

**Published:** 2019-03-14

**Authors:** Gang Liu, Yahong Wang, Lawei Yang, Baoan Zou, Shenglan Gao, Zeqing Song, Ziying Lin

**Affiliations:** ^1^ Shenzhen Longhua District Central Hospital Shenzhen China; ^2^ Clinical Research Center Affiliated Hospital of Guangdong Medical University Zhanjiang China; ^3^ Department of Respiratory Medicine Affiliated Hospital of Guangdong Medical University Zhanjiang China

**Keywords:** AECs, beta‐catenin, EMT, IPF, Smad2/3, TSPAN1

## Abstract

Tetraspanin 1(TSPAN1) as a clinically relevant gene target in cancer has been studied, but there is no direct in vivo or vitro evidence for pulmonary fibrosis (PF). Using reanalysing Gene Expression Omnibus data, here, we show for the first time that TSPAN1 was markedly down‐regulated in lung tissue of patient with idiopathic PF (IPF) and verified the reduced protein expression of TSPAN1 in lung tissue samples of patient with IPF and bleomycin‐induced PF mice. The expression of TSPAN1 was decreased and associated with transforming growth factor‐β1 (TGF‐β_1_)‐induced molecular characteristics of epithelial‐to‐mesenchymal transition (EMT) in alveolar epithelial cells (AECs). Silencing TSPAN1 promoted cell migration, and the expression of alpha‐smooth muscle actin, vimentin and E‐cadherin in AECs with TGF‐β_1_ treatment, while exogenous TSPAN1 has the converse effects. Moreover, silencing TSPAN1 promotes the phosphorylation of Smad2/3 and stabilizes beta‐catenin protein, however, overexpressed TSPAN1 impeded TGF‐β_1_‐induced activation of Smad2/3 and beta‐catenin pathway in AECs. Together, our study implicates TSPAN1 as a key regulator in the process of EMT in AECs of IPF.

## 1 INTRODUCTION

Pulmonary fibrosis (PF) is a chronic lung disease involving pulmonary injury associated with tissue repair, dysfunction and fibrosis. Idiopathic PF (IPF) is considered the most common and severe form of the disease with no proven effective therapy, however, the precise mechanisms that drive fibrosis in most patients remain incompletely understood.^1^, ^2^ Capitalizing on the evolving understanding of IPF biology to develop new targets will facilitate an accurate diagnostic and therapy process. Idiopathic PF is often associated with abnormal tissue repair caused by inflammation, fibroblast proliferation and excessive production of extracellular matrix (ECM).^3^ It is well known that myofibroblasts are the main effector cells in the pathogenesis of PF.^4^, ^5^ Activated fibroblasts and myofibroblasts are implicated as influential in the process of fibrogenesis, including that of PF.^2^ In the lung, myofibroblasts are mainly derived from activated lung fibroblasts.^6^, ^7^ Upon lung injury, non‐contractile fibroblasts convert into activated myofibroblasts that express alpha‐smooth muscle actin (α‐SMA). Accumulating evidences have demonstrated that alveolar epithelial cells (AECs) injury leads to aberrant activation of AECs, losing their normal epithelial regenerative capacity, executing epithelial‐to‐mesenchymal transition (EMT), which resulting in AECs transformed to migratory and/or invasive mesenchymal cells (fibroblast‐like spindle cell morphology), creating a profibrotic environment with accumulation of collagen‐producing fibroblasts and myofibroblasts.^8^, ^9^ Transforming growth factor‐β (TGF‐β) signalling is a powerful inducer of EMT, mostly through its canonical Smad‐dependent pathway.^10^, ^11^ Transforming growth factor‐β signals through type I and type II serine/threonine kinase receptors, phosphorylating Smad2 and Smad3.^11^ In addition, some reports have implicated interactions between TGF‐β and β‐catenin signalling pathways in EMT, and β‐catenin binds Smad3 and cAMP response element‐binding and (CREB)‐binding protein (CBP) to release myocardin‐related transcription factor (MRTF) from Smad3, allowing MRTF to activate the α‐SMA promoter.^12^, ^13^, ^14^ Therefore, It is considered that TGF‐β‐induced EMT of AECs may dependent crosstalk between Smad2 and β‐catenin signaling pathways in process of IPF.

Tetraspanins, a family which have four transmembrane (TM) domains, can form massive protein‐protein complexes that transduce extracellular signals to intracellular signalling pathways on the surface of cells.^15^ Some reports confirmed that they can affect EMT to mediate fibrotic phenotypes by forming tetraspanin‐enriched membrane micro‐domains (TEMs) with a variety of transmembrane and cytosolic proteins.^16^, ^17^, ^18^ Tetraspanin 1 (TSPAN1), which is one of the tetraspanin family member, plays multiple roles in human cancer, involving cell‐cycle, proliferation, migration and invasion.^19^, ^20^, ^21^ Surprisingly, we found that TSPAN1 was an abnormal gene through IPF patient Gene Expression Omnibus (GEO) online data analysis. Furthermore, we demonstrated that the protein expression of TSPAN1 was significantly decreased during TGF‐β_1_‐induced cell treatment in A549 and primary rat alveolar epithelial type II (ATII) cells. And it was verified in the lung section of IPF patients and bleomycin‐model of PF. Therefore, we tried to explore the possible mechanism based on the TSPAN1‐mediated EMT in IPF by the molecular biology approach.

## 2 MATERIALS AND METHODS

### 2.1 Reagents

Recombinant Human TGF‐β_1_(100‐21) was obtained from PeproTech (Rocky Hill, NJ, USA). The antibodies used were as follows: E‐cadherin (1:1000, #14472), N‐cadherin (1:1000, #14255), Vimentin (1:1000, #5741), Smad2/3(1:1000, #5678), p‐Smad2/3 (Ser465/467/ser423/425, 1:1000, #8828), beta‐catenin (1:1000, #9562) were purchased from Cell Signaling Technology (CST, Danvers, MA). Transforming growth factor‐β (1:1000, sc‐146) and α‐SMA (1:1000, sc‐53015) were purchased from Santa Cruz Biotechnology (Dallas, TX, USA). FITC Mouse Anti‐Human CD51/CD61 (555505; BD biosciences, San Jose, CA, USA) and PE Mouse Anti‐Human CD106 were obtained from BD biosciences. Tetraspanin 1 (1:200, NBP2‐33867) was purchased from Novus Biologicals (LLC, Littleton Co., Centennial, CO, USA). Bleomycin was obtained from Sigma (St. Louis, MO).

### 2.2 GEO data overview

The transcription profile of GSE32539^13^ (a total of 169 samples, including 119 samples collected from patients with IPF/UIP and 50 non‐diseased lung tissues) was obtained from NCBI GEO database (http://www.ncbi.nlm.nih.gov/geo/) which is based on Affymetrix microarray platforms (Human Gene 1.0 ST Array). The format of dataset was transformed and normalized and applied to identify genes which were significantly differentially expressed between disease samples and normal control. Eleven‐thousand nine hundred and fifty transcripts were retained in the dataset. More information about this GEO data as described in this report.^22^ We defined *P*‐value <0.05 to be statistically significant. Differentially expressed analysis was performed by Shanghai Biotechnology co. (Shanghai, China).

### 2.3 Cell culture and treatment

Human AECs A549 and lung fibroblasts cells MRC‐5 were obtained from Shanghai Cell Institute Country Cell Bank (Shanghai, China). A549 and MRC‐5 cells were grown in DMEM, which containing 10% fetal bovine serum (FBS), 100 U/mL penicillin and 100 μg/mL streptomycin. Cells were cultured at 37°C in a humidified 5% CO_2_ incubator. Primary rat ATII cells and rat fibroblasts were isolated from the lungs of neonatal rats within 24 hours of birth. The isolation and culture of primary rat ATII cells were performed, as described previously.^23^ Purified ATII cells and fibroblasts were cultured in DMEM‐F12 medium (Gibco, Waltham, MA) with 10% FBS for subsequent experiments. The ATII cells were identified by immunofluorescence staining with surfactant protein‐C (SP‐C) and transmission electron microscopy (TEM). Primary rat fibroblasts were identified by immunofluorescence staining with α‐SMA. For TGF‐β_1_ treatment, all cells were serum starved in 1% FBS overnight prior to stimulation with 5 ng/mL recombinant TGF‐β_1_ for 12 or 24 hours.

### 2.4 Patient

Normal lung tissue samples (paracancerous tissue) were obtained from the Department of Cardiothoracic Surgery, Affiliated Hospital of Guangdong Medical University. Pulmonary fibrosis tissue samples were collected from Nanjing Drum Tower Hospital, the affiliated hospital of Nanjing University Medical School. This study was approved by the Affiliated Hospital of Guangdong Medical University Ethics Committee (No: PJ2012132), and carried out under approved guidelines. Patients were told that lung tissue from them were used for medical research and confirmed ‘informed consent' for this project. The clinical characteristic of the patients was described in Table 1.

**Table 1:** The clinical and pathological characteristics of patients

Patient number	Sample type	Age	Gender	Smoking	FVC (%)	FEV_1_ (%)	DLCO (%)
1	N	54	Female	Yes	/	/	/
2	N	71	Male	Yes	/	/	/
3	N	65	Male	No	/	/	/
4	IPF	66	Male	Yes	44.6	52.6	14.6
5	IPF	57	Male	Yes	48.9	52.9	15.6
6	IPF	74	Male	Yes	64.3	52.9	35.5

DLCO, diffusion capacity for carbon monoxide; FEV_1_, forced expiratory volume in 1 sec; FVC, forced vital capacity; IPF, idiopathic pulmonary fibrosis; N, normal lung (paracancerous).

### 2.5 Bleomycin‐model of PF

All animal experiments were carried out in accordance with ethical guidelines from Guangdong Medical University Ethics Committee of Animal Care. In this study, 6 to 8‐week‐old and 18‐25 g C57BL/6 mice were anesthetized with ketamine/xylazine (ip, 23 mg/kg). Bleomycin or saline was instilled intratracheally at a dose of 3.5 U/kg to mice in 0.1 mL saline by. After 21 days, the model mice were considered to reflect the peak of fibrosis, mice were killed and the samples were removed for experiments.

### 2.6 Overexpression and knockdown of TSPAN1 in lung cells

The full length of human TSPAN1 was cloned into pCDNA3.1 + vector (Promega, USA). Expression of TSPAN1 in A549 cells was silenced using specific TSPAN1 siRNA [TSPAN1 siRNA‐1 and TSPAN1 siRNA‐2 (GenePharma company, China)]. Scramble siRNA negative control for siRNA (NC‐siRNA) was used to confirm the specificity of TSPAN1 siRNAs. The TSPAN1 siRNA‐1 group sequences were 5ʹ‐CCAUGAUGAUCCUCUUCAATT‐3ʹ (sense) and 5ʹ‐UUGUGGUGUACACCAAGGCTT‐3ʹ (antisense). The TSPAN1 siRNA‐2 group sequences were 5ʹ‐GCCUUGGUGUACACCACAATT‐3ʹ (sense) and 5ʹ‐UCGGAUGUCACACCAAGGCTT‐3ʹ (antisense). The scramble siRNA group sequences were 5ʹ‐UUCUCCGAACGUGUCACGUdTdT‐3ʹ (sense) and 5ʹ‐ACGUGACACGUUCGGAGAAdTdT‐3ʹ (antisense). Transfection was performed using Lipofectamine 2000 (Invitrogen, Carlsbad, CA, USA) according to the manufacturer's protocol.

### 2.7 Western blotting

The tissues and cells were lysed on ice in lysis buffer (Beyotime, China) supplemented with 1 mmol/L phenylmethanesulfonyl fluoride (Sigma), and centrifuged at 8049.6 *g* for 15 minutes at 4°C. According to standard Western blot procedures, every group of protein was pooled for 10%‐12% SDS‐PAGE. After blocking in 5% non‐fat milk, the membranes were incubated with the following primary antibodies. The proteins were visualized with enhanced chemiluminescence reagents (Pierce, Waltham, MA, USA ). Western blot was quantified using ImageJ analysis of scanned blots.

### 2.8 Transwell assays

Cell migration potential was evaluated using transwell chambers (8 μm pore; BD Biosciences). Briefly, 5 × 10^4^ transfected cells were suspended in 100 µL of DMEM medium and placed into the upper side of the polycarbonate transwell filter. The lower chambers were filed with 600 µL of medium containing 10% FBS as inducer. After incubation for 24 hours in a humidified atmosphere of 5% CO_2_ at 37°C, cells on upper chamber were removed from with a cotton swab, while migrated cells were fixed with 70% ethanol for 30 minutes and stained with 0.2% crystal violet for 1 hour. Photographs of 5‐8 randomly selected fields of the fixed cells were taken and counted under an inverted light microscope (Leica, German).

### 2.9 Wound healing assays

Cells were seeded into six‐well plates and cultured in DMEM medium with 10% FBS. Then, cells were starved overnight in serum‐free DMEM. A straight wound was created using a sterile 200 µL pipette tip, and the cells were washed with PBS to remove floating cells and debris and smooth the edge of the scratch. At 0 and 24 hours, images were taken on a microscope (Leica, German). The experiments were performed in duplicate and repeated three times. Migration ability was assessed by measuring changes in the width of the wounded areas.

### 2.10 Analysis of gene expression

Total RNA was extracted from samples using the TRIzol reagent (Ambion^®^) according to the manufacturer's protocol. RNA samples were then reverse transcribed into cDNA, using a FastQuant RT Kit (with gDNase) (Tiangen, China) in a total volume of 20 μL according to the manufacturer's protocol. Equal amounts of cDNA samples were used as a template for real‐time PCR to detect the level of TSPAN1 expression. glyceraldehyde‐3‐phosphate dehydrogenase (GAPDH) was used as an endogenous reference, and each sample was normalized to its GAPDH content. Primer sequences used are shown in Table 2. All experiments were performed in duplicate and repeated three times. Results represented the fold induction using the 2^−ΔΔCt^method.

**Table 2:** Primer sequences for reverse transcription quantitative real‐time polymerase chain reaction

Gene	Forward	Reverse
Cdh2	CACAACGGACTATGAAACAC	GCTGATAAACTTCGGGTC
Pdgfra	GAAAGGCAAAGGCATCAC	CCGCACCTCTACAACAAA
Pdgfrb	GGTGGATTCTGATGCCTACT	ATGGAGCGGATGTGGTAA
Zeb1	GTGGCGGTAGATGGTAAT	TGTTGTATGGGTGAAGCA
TSPAN1	CTCAAGTGCTGTGGCTTCAC	AGGTTTCATTGGCTGTGTTG
Collagen I	GCGAGAGCATGACCGATGGATTC	GCCTTCTTGAGGTTGCCAGTCTG
GAPDH	AGAAGGCTGGGGCTCATTTG	AGGGGCCATCCACAGTCTTC

### 2.11 Immunofluorescence assays

Cells were fixed in 4% formaldehyde on coverslips for 15 minutes, followed by incubation in PBS solution supplemented with 1% Triton X‐100 (PBS‐Triton) and 2.5% bovine serum albumin (BSA) for blocking for 20 minutes. Then cells were incubated with primary antibodies at dilution of 1:50‐100 (for all the primary antibodies used) in 2.5% BSA at 4°C and overnight, followed by five washes with PBS solution. Cells were then incubated with secondary antibodies pre‐conjugated at dilution of 1:100 (for both secondary antibodies) in 2.5% BSA for 30 minutes at room temperature, followed by five washes with PBS solution. The nuclei were stained with 4′,6‐diamidino‐2‐phenylindole (DAPI). Images were recorded with a confocal microscope (TCS SP5 II; Leica, Wetzlar, Germany).

### 2.12 Flow cytometry analysis

The expression of the cell surface markers CD51/CD61 and CD106 on A549 cells was analyzed using flow cytometry. Briefly, cells with or without TGF‐β_1_ treatment for 24 hours were suspended in PBS containing 2% BSA (10^4^ cells/µL). Combinations of FITC‐CD51/CD61 and PE‐CD106 or their respective isotype control antibodies were added to cell suspensions at the concentrations recommended by the manufacturer and then incubated at 4°C in the dark for 30 minutes. The labelled cells were washed with PBS and then analysed on a FACSCanto II flow cytometer (BD Biosciences).

### 2.13 Statistical analysis

Data were shown as mean ± SEM unless otherwise noted. The two‐tailed Student's *t* test was used to analyse the difference between two groups for cells and non‐parametric tests were to analyse the difference between two groups for tissues. The *P* value of <0.05 was considered statistically significant.

## 3 RESULTS

### 3.1 Differential expression analyses reveal expression of TSPAN1 was reduced in IPF

**Figure 1:**
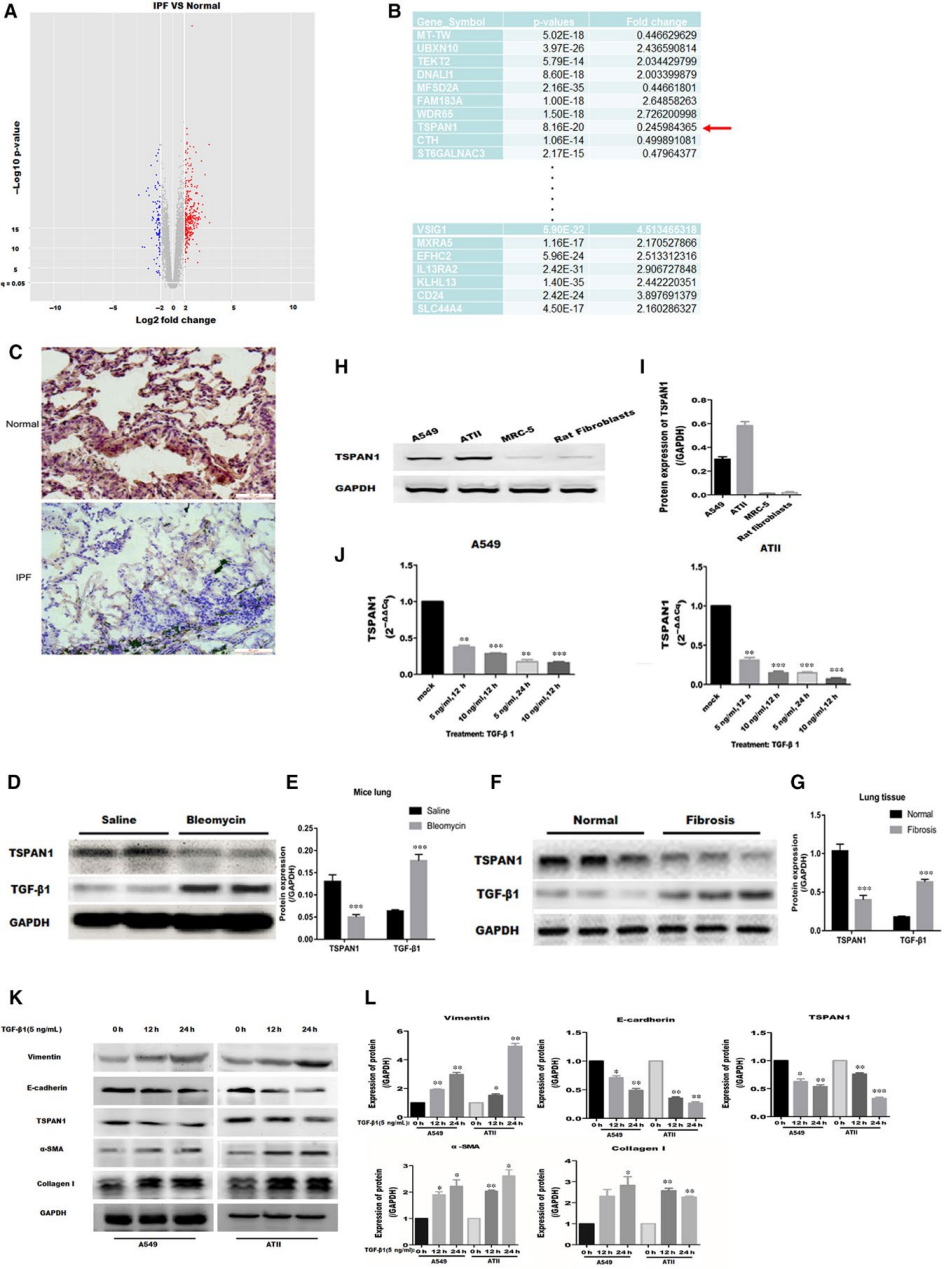
Screening of gene in idiopathic pulmonary fibrosis (IPF) and identification of tetraspanin 1 (TSPAN1) in vivo and *vitro*. A, Volcano plot shows that the two vertical lines are the two‐fold change boundaries and the horizontal line is the statistical significance boundary (*P* < 0.05). Genes with fold change ≥2 and statistical significance are marked with red dots, genes with fold change ≤0.5 and statistical significance are marked with blue dots. B, tetraspanin 1 as a candidate gene was low expressed in the IPF and the fold change was marked by red arrow. C, The protein expression of TSPAN1 in IPF and normal lung tissue was detected by immunohistochemical staining. Magnification: 200X. D, All specimens were observed from mice at day 21 after bleomycin treatment, and the protein expression of TSPAN1 and transforming growth factor‐β1 (TGF‐β_1_) was verified by Western blotting in the mouse model of bleomycin‐induced pulmonary fibrosis. E, Quantification results in panel D. The data are expressed as the means ± SEM (n = 3). Representative data from 1 of three independent experiments are shown (***P < 0.001 vs Saline). F, Lung tissue of IPF or normal tissues were obtained after surgery. Protein expression of TSPAN1 and TGF‐β_1_ was detected by Western blotting in Lung tissue of IPF or normal tissue. G, Quantification of the results in panel F. The data are expressed as the means ± SEM (n = 3) (*P < 0.05 vs normal). H, Western blotting was used to detect the protein expression of TSPAN1 in A549, alveolar epithelial type II (ATII) cells, MRC‐5 and rat fibroblasts. I, Quantification of the results in panel H. The data are expressed as the means ± SEM (n = 3), with results representative of three independent experiments. J, A549 and ATII cells were treated with TGF‐β_1_ (5 or 10 ng/mL) for 12 or 24 h and then the mRNA expression of TSPAN1 was detected by quantitative real‐time PCR in the cells, data are expressed as the means ± SEM (n = 3), with results representative of three independent experiments. (*P < 0.05, **P < 0.01 and ***P < 0.001 vs mock). K, A549 and ATII cells were treated with TGF‐β_1_ (5 ng/mL) for 12 or 24 h and the protein expression of TSPAN1 and epithelial‐to‐mesenchymal transition‐related marker were detected by Western blotting in the cells. L, Quantification of the results in panel (K). The data are expressed as the means ± SEM (n = 3), with results representative of three independent experiments. (*P < 0.05, **P < 0.01 and ***P < 0.001 vs 0 h)

Aberrant genes expression was found by analysing the raw data of GSE32539 in lung tissue from IPF patients compared to the normal lung tissue. Here, 339 genes were up‐regulated more than two‐fold and 102 genes were down‐regulated less than 0.5‐fold in PF, in comparison with normal lung tissue. A volcano plot of the identified quality‐controlled genes (*P* < 0.05, fold change ≥2 or ≤0.5) is presented in Figure 1A. Then, we selected a part of differential expression genes to be further verified as candidate genes. In these genes, TSPAN1 was a down‐regulated gene in IPF (Figure 1B, *P* < 0.05, fold change <0.5).

### 3.2 Validation of TSPAN1 expression *in vivo* or *vitro*

To identify the expression level of TSPAN1, we observed the expression of TSPAN1 in lung tissue of bleomycin‐induced PF mice and IPF patients. Firstly, we obtained lung tissues from mice with or without bleomycin‐induced PF and detected the expression of TSPAN1 in lung tissues. We found the expression of TSPAN1 was also decreased in fibrotic lung tissue from mice (Figure 1D,E, *P* < 0.001). Then, we detected the protein expression of TSPAN1 in lung tissues of patients with IPF compared to normal lung tissues. Immunohistochemical staining and Western blot analysis demonstrated that TSPAN1 was decreased in lung tissues from patients with IPF (Figure 1C,F and G, *P* < 0.05). Transforming growth factor‐β1 is up‐regulated in the lung tissue of bleomycin‐induced PF in mice and patients with IPF (Figure 1D‐G, *P* < 0.05). Moreover, the protein expression of TSPAN1 was detected in AECs (A549 and ATII cells) and lung fibroblasts (MRC‐5 and Rat fibroblasts) without any treatment, and Western blot analysis showed TSPAN1 was high‐expressed in AECs and low‐ expressed in lung fibroblasts (Figure 1H,I). In addition, the result showed that mRNA expression analysis of TSPAN1, using quantitative real‐time PCR (QPCR) was significantly decreased under TGF‐β_1_‐treatment in AECs and showed a dose‐dependent and time‐dependent tendency (Figure 1J, *P* < 0.05) and Western blot analysis was used to detect protein expression of TSPAN1 and EMT‐related, see Figure 1K and L, after TGF‐β_1_ induced cells for 12 and 24 hours at 5 ng/mL, TSPAN1 and E‐cadherin were reduced, Vimentin,α‐SMA and collagen I was up‐regulated in A549 and ATII cells. These data confirmed that TSPAN1 was reduced in PF and fibroblasts‐like cells.

### 3.3 Knockdown TSPAN1 promoted molecular characterization of EMT state in A549

**Figure 2:**
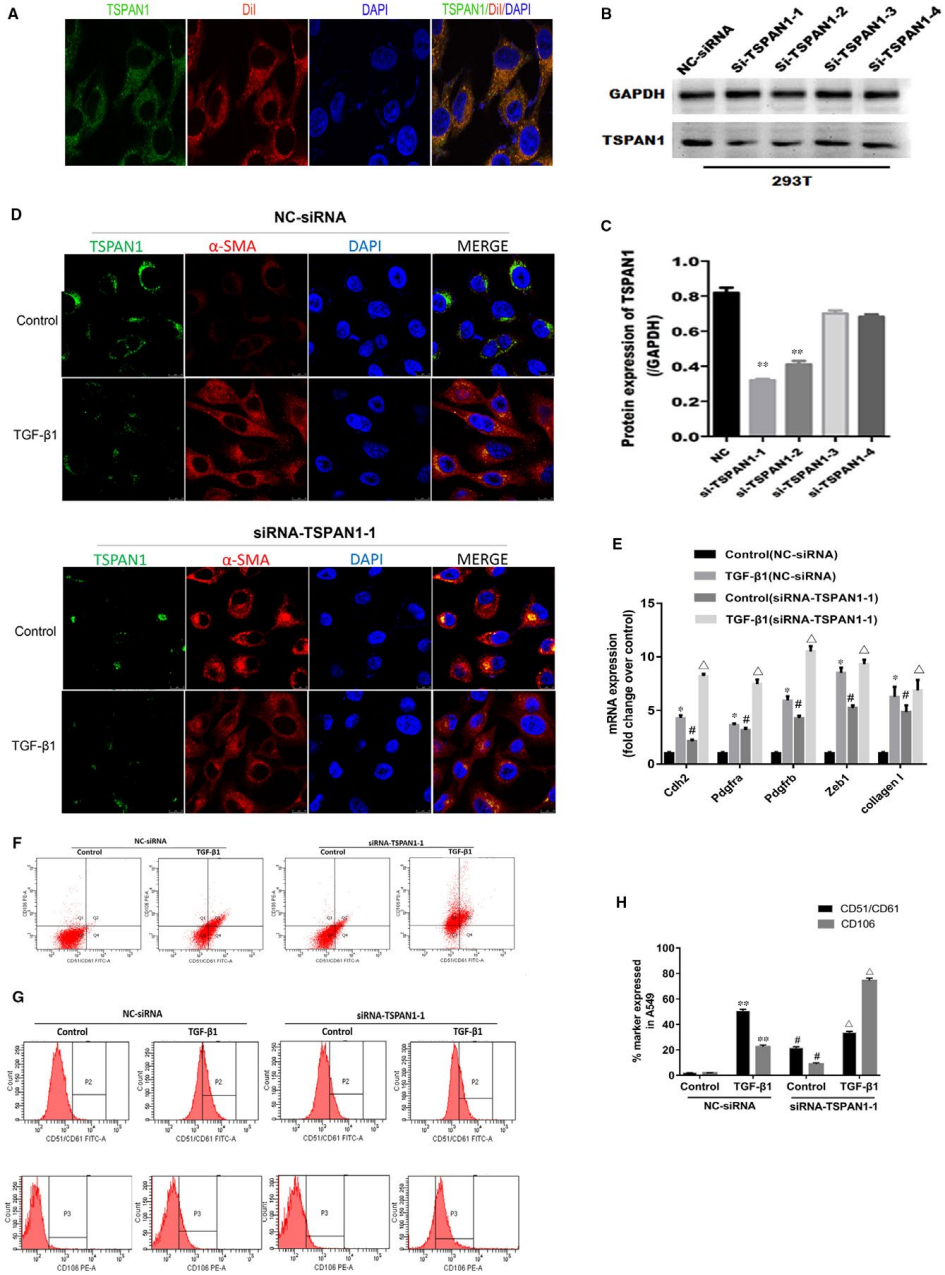
Knockdown tetraspanin 1 (TSPAN1) promoted molecular characterization of epithelial‐to‐mesenchymal transition state in alveolar epithelial cells. A, Immunofluorescence analysis of subcellular localization for TSPAN1 in A549 cells (green: TSPAN1; red: DiI‐cell membrane red fluorescent probe; blue: 4′,6‐diamidino‐2‐phenylindole [DAPI]), and TSPAN1 was co‐located with DiI in A549 cell. Magnification: 1260X. B, Interference efficiency of siRNA for TSPAN1 was identified in 293T cells by Western blotting. Four siRNA sequences, named si‐TSPAN1‐1, si‐TSPAN1‐2, si‐TSPAN1‐3 and si‐TSPAN1‐4, were designed to aim four target sites for TSPAN1. C, Quantification of the results in panel (B). The data are expressed as the means ± SEM (n = 3), with results representative of three independent experiments. (**P < 0.01 vs negative control for siRNA [NC‐siRNA]). (D) Immunofluorescence staining for TSPAN1 and alpha‐smooth muscle actin (α‐SMA) after transfected with siRNA for TSPAN1 or NC in A549 cells with or without TGF‐β_1_treatment (5 ng/mL TGF‐β_1_or control) (Green: TSPAN1; Red: α‐SMA; Blue: DAPI). Magnification: 1200X. E, mRNA expression of mesenchymal genes in A549 with or without TGF‐β_1_ treatment as defined by qRT‐PCR. The data are expressed as the means ± SEM (n = 3), with results representative of three independent experiments. (*P < 0.05, **P < 0.01, ***P < 0.001 vs control; *^#^P* < 0.05, *^##^P* < 0.01, *^###^P* < 0.001 vs control [NC‐siRNA]; Δ*P* < 0.05, ΔΔ*P* < 0.01, ΔΔΔ*P* < 0.001 vs control [siRNA‐TSPAN1‐1]). (F,G) Fluorescence activated cell sorting profile showed the CD51/CD61 and CD106 subpopulation in A549 cells with or without TGF‐β_1_ treatment. H, Quantification of the results in panel (F) and (G). The data are expressed as the means ± SEM (n = 3), with results representative of three independent experiments. (*P < 0.05, **P < 0.01, ***P < 0.001 vs control; ^#^ *P* < 0.05, ^##^ *P* < 0.01, ^###^ *P* < 0.001 vs control [NC‐siRNA]; Δ*P < *0.05, ΔΔ*P* < 0.01, ΔΔΔ*P < *0.001 vs control [siRNA‐TSPAN1‐1])

We investigated the role of TSPAN1 on molecular characterization of EMT. The localization of TSPAN1 in A549 cells was examined. Co‐immunofluorescence staining showed that TSPAN1 is co‐localized with the membrane probe‐DiI, indicating that TSPAN1 is localized on the cell membrane surface (Figure 2A). Four siRNA designed to knockdown TSPAN1 were checked in 293T cells. As shown in Figure 2B and C, the expression of TSPAN1 by Western blot analysis was obviously decreased in si‐TSPAN1‐1 and si‐TSPAN1‐2 group, compared to NC‐siRNA group (Figure 2B and C, *P* < 0.01). Therefore, si‐TSPAN1‐1 and si‐TSPAN1‐2 were selected in subsequent studies. Next, we used the two siRNA to knockdown the expression of TSPAN1 for ensuring the effect of siRNA in A549 cells. And then, we also detected the expression of α‐SMA by immunofluorescence after TSPAN1‐knockdown in A549 cells, TGF‐β_1_ increased the expression of α‐SMA and inhibited the expression of TSPAN1, silencing TSPAN1 promoted the expression of α‐SMA induced by TGF‐β_1_ in A549 cells (Figure 2D). In addition, mRNA expression of mesenchymal genes following TSPAN1‐knockdown in A549 with or without TGF‐β_1_ treatment was detected by QPCR. The mRNA expression of Cdh2, Pdgfa, Pdgfb, Zeb1 and collagen I were increased in TGF‐β_1_‐treated A549 cells compared with untreated A549 cells. Meantime, we found the mRNA expression of Cdh2, Pdgfa, Pdgfb, Zeb1 and collagen I were also increased in TSPAN1 knockdown A549 cells without TGF‐β_1_ treatement (Figure 2E). Finally, we analysed the effect of TSPAN1 on cell surface markers of EMT‐CD51/CD61 and CD106 subpopulation by flow cytometry (Figure 2F,G). The results by flow cytometry showed that the expression of CD51/CD61 and CD106 were increased in TGF‐β_1_‐treated A549 compared with untreated A549, and the expression of CD51/CD61 and CD106 were increased in TSPAN1 knockdown A549 cells (Figure 2H).

### 3.4 Silencing TSPAN1 enhanced cell migration and TGF‐β_1_‐induced EMT‐related proteins expression in alveolar epithelial cells

**Figure 3:**
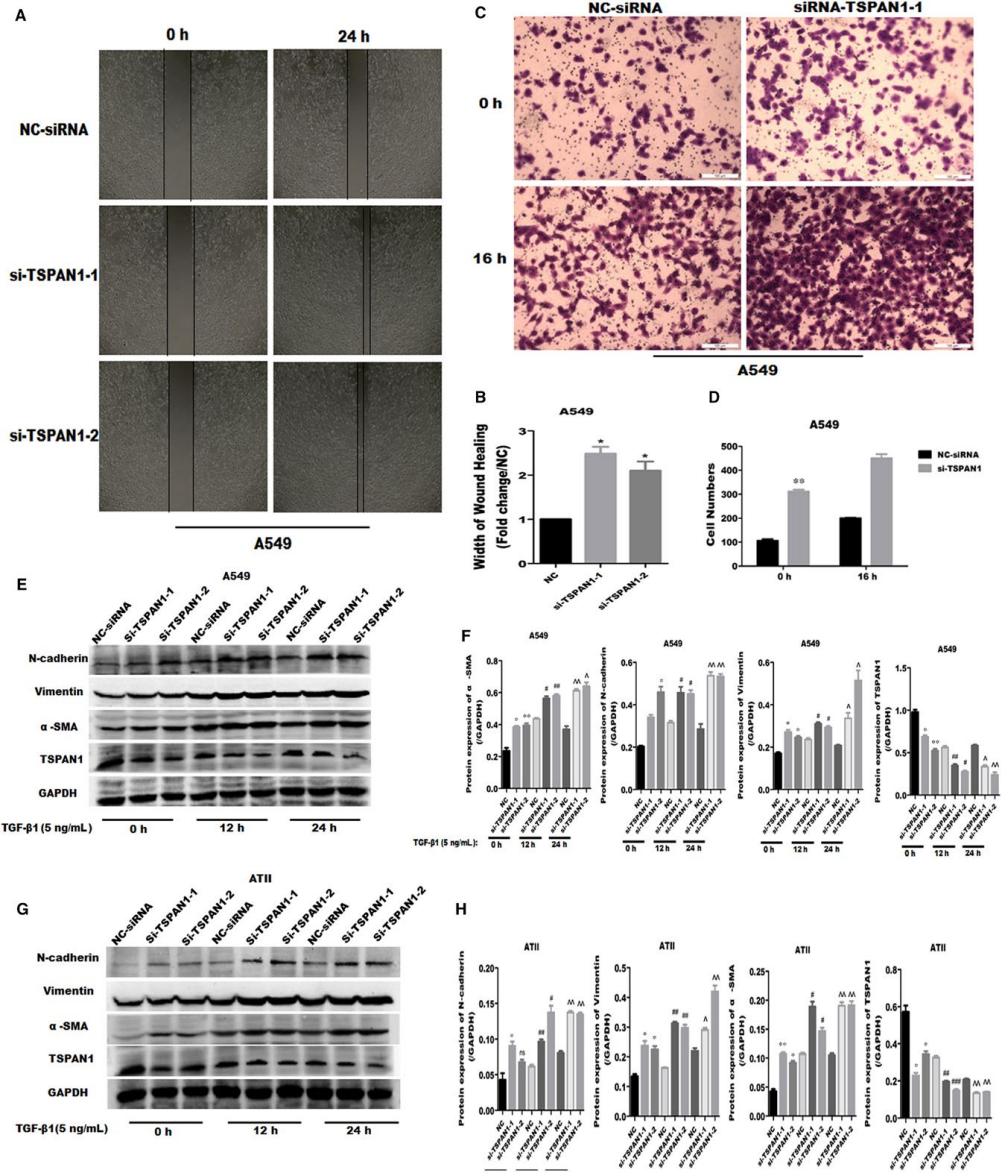
Knockdown tetraspanin 1 (TSPAN1) promoted cell migration and transforming growth factor‐β1 (TGF‐β_1_)‐induced epithelial‐to‐mesenchymal transition‐related proteins in alveolar epithelial cells. A, Wound healing assay following silencing of TSPAN1 in A549 cells. B, Quantification results of wound healing assay. Bar chart represents the ratio of every group to negative control (NC) groups. The data are expressed as the means ± SEM (n = 3), with results representative of three independent experiments. (*P < 0.05 vs NC). C, Transwell migration analysis by silencing TSPAN1 in A549 cells with or without TGF‐β_1_ treatment (5 ng/mL, 16 h). D, Quantification of transwell migration analysis. The bar chart represents the numbers of migrating cells. The data are expressed as the means ± SEM (n = 3), with results representative of three independent experiments. (**P < 0.01 vs NC). (E,G) Western blot analysis of various proteins by silencing TSPAN1 for 24 h in A549 and alveolar epithelial type II cells with or without TGF‐β_1_ treatment. F, Quantification of the results in panel (E), and (H) quantification of the results in panel (G). The data are expressed as the means ± SEM (n = 3), with results representative of three independent experiments. (*P < 0.05, **P < 0.01, ***P < 0.001 vs NC 0 h; ^#^ *P < *0.05, ^##^ *P < *0.01, ^###^ *P < *0.001 vs NC 12 h; Δ*P < *0.05, ΔΔ*P < *0.01, ΔΔΔ*P* < 0.001 vs NC 24 h)

We further investigated the role of TSPAN1 in cell migration and TGF‐β_1_‐induced EMT‐related proteins. Wound healing and transwell assays were used to examine the effect of silencing TSPAN1 on cell migration. As shown in Figure 3A, the narrower width was evaluated after TSPAN1‐knockdown (transfected with si‐TSPAN1‐1 and si‐TSPAN1‐2) in A549 cells compared to negative control (NC; Figure 3B, *P* < 0.05). Similarly, the number of cells invaded across the polycarbonate membrane was significantly increased in knockdown TSPAN1 (transfected with si‐TSPAN1‐1) in A549 cells with or without TGF‐β_1_ treatment, compared to NC (Figure 3C and D, *P* < 0.01). These results indicate TSPAN1‐knockdown induced cell migration in A549 cells. Moreover, we detected the expression of EMT‐associated factors after TSPAN1‐knockdown in A549 and ATII cells by Western blot analysis, the protein expression of N‐cadherin, vimentin and α‐SMA was increased in knockdown TSPAN1 in A549 (Figure 3E,F) and ATII cells (Figure 3G,H) with or without TGF‐β_1_ treatment. Taken together, these results indicate silencing TSPAN1 could enhance cell migration by promoting EMT in AECs.

### 3.5 Exogenous expression of TSPAN1 impeded TGF‐β_1_‐induced EMT and migration in alveolar epithelial cells

**Figure 4:**
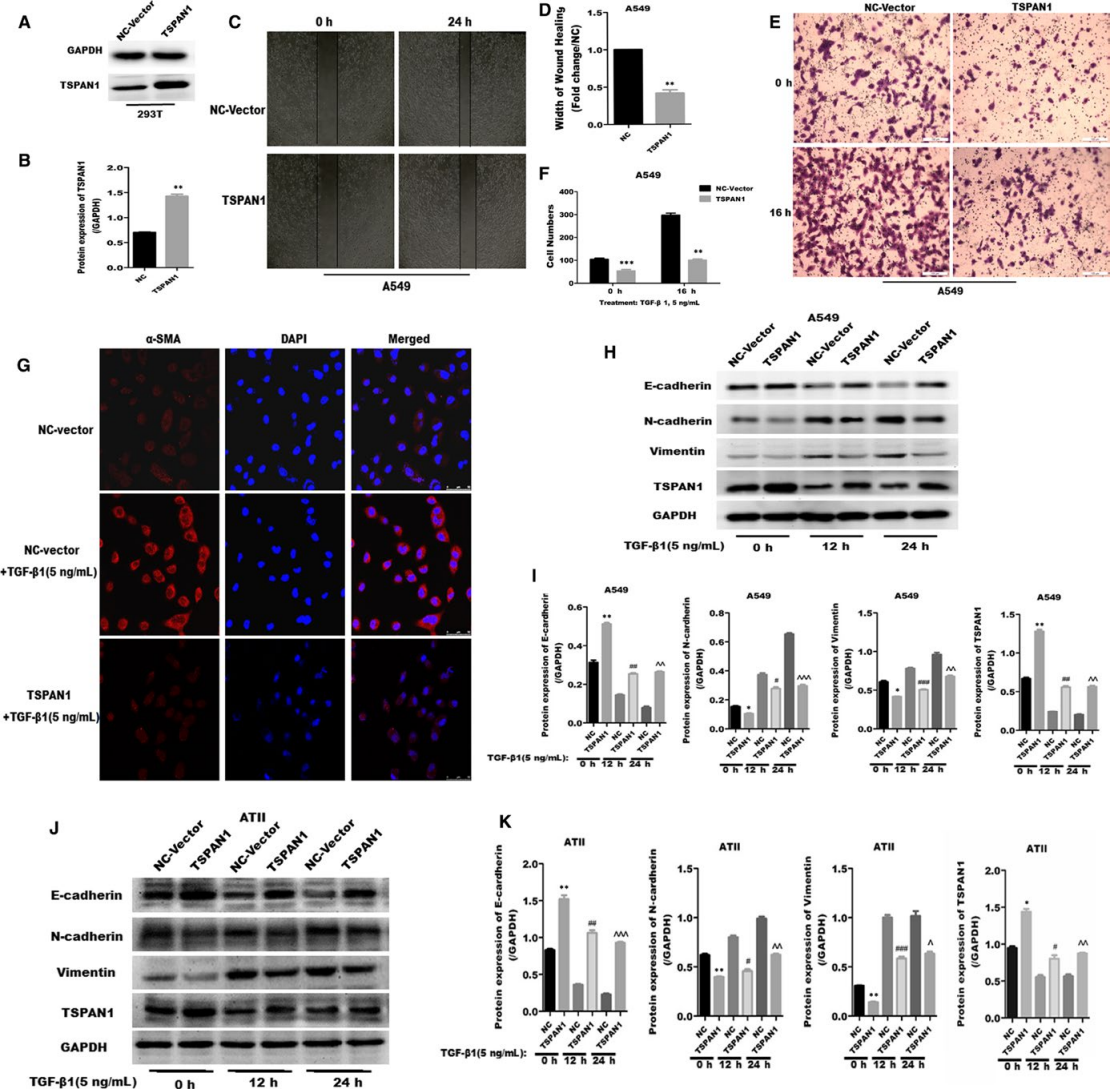
Inhibition of cell migration and transforming growth factor‐β1 (TGF‐β_1_)‐induced epithelial‐to‐mesenchymal transition by tetraspanin 1 (TSPAN1) overexpression in alveolar epithelial cells. A, Efficiency of exogenous expression for TSPAN1 was determined by Western blotting in 293T cells. B, Quantification of the results in panel (A). The data are expressed as the means ± SEM (n = 3), Results are representative of at least three independent experiments. (**P < 0.01 vs negative control [NC]). C, Wound healing analysis by overexpression of TSPAN1 in A549 cells. D, Quantification of wound healing. Bar chart represents the ratio of every group to NC groups. The data are expressed as the means ± SEM (n = 3), results are representative of three independent experiments. (**P < 0.01 vs NC). E, Transwell migration analysis after overexpression of TSPAN1 in A549 cells with or without TGF‐β_1_ treatment (5 ng/mL, 16 h). F, Quantification of transwell migration analysis. Bar chart represents the numbers of migrating cells. The data are expressed as the means ± SEM (n = 3), results are representative of three independent experiments. (**P < 0.01, ***P < 0.001 vs NC). G, The expression of alpha‐smooth muscle actin (α‐SMA) (red fluorescence) was detected by immunofluorescence. Transforming growth factor‐β1‐induced the expression of α‐SMA, and red fluorescence was increased; However, Overexpression of TSPAN1 inhibited exaltation of red fluorescence induced by TGF‐β_1_ in A549 cells. Magnification: 630X. (H,J)Western blot analysis of various proteins after overexpression of TSPAN1 for 24 h in A549 and alveolar epithelial type II cells with or without TGF‐β_1_ treatment. I, Quantification of results in panel (H), and (K) quantification of results in panel (J). The data are expressed as the means ± SEM (n = 3), with results representative of three independent experiments. (*P < 0.05, **P < 0.01, ***P < 0.001 vs NC 0 h; ^#^ *P < *0.05, ^##^ *P < *0.01, ^###^ *P < *0.001 vs NC 12 h; Δ*P < *0.05, ΔΔ*P* < 0.01, ΔΔΔ*P < *0.001 vs NC 24 h)

Correspondingly, we also verified the affinity of TSPAN1‐overexpression on EMT. We transfected with exogenous expression of TSPAN1 or NC vector in A549 or ATII cells. At first, we transfected exogenous expression plasmid of TSPAN1 and NC vector named NC‐Vector into 293T cells. After 24 hours, the protein expression of TSPAN1 was evaluated by Western blot analysis and the expression of TSPAN1 was significantly increased in TSPAN1 groups compared to NC‐Vector groups (Figure 4A and B, *P* < 0.01). The results certified the expression vector of TSPAN1 was effectively transcribed and translated into 293T cells.

Then, wound healing and transwell assays were used to examine the effect of exogenous‐expressed TSPAN1 on cell migration. Wound healing assay showed the wider width was founded in overexpressed TSPAN1 in A549 cells, compared to NC (Figure 4 C and D, *P* < 0.01). On the other hand, transwell analysis showed the number of cells invaded across the polycarbonate membrane was significantly decreased in overexpressed TSPAN1 in A549 cells with or without TGF‐β_1_ treatment, compared to NC (Figure 4E,F). And then, we also detected the protein expression of α‐SMA by immunofluorescence after TSPAN1‐overexpression in A549 and ATII cells. Transforming growth factor‐β1 increased the expression of α‐SMA, however, exogenous expressed TSPAN1 inhibited the exaltation of α‐SMA induced by TGF‐β_1_ in A549 cells. Western blot analysis showed the expression of N‐cadherin, Vimentin were increased and the E‐cadherin was decreased in overexpressed‐TSPAN1 in A549 (Figure 4H‐I) and ATII cells (Figure 4J‐K) with or with TGF‐β_1_ treatment for 12 or 24 hours. Therefore, these results indicate that exogenous expression of TSPAN1 could block TGF‐β_1_‐induced EMT and cell migration in AECs.

### 3.6 TSPAN1 regulated beta‐catenin and phosphorylation of Smad2/3 in alveolar epithelial cells

**Figure 5:**
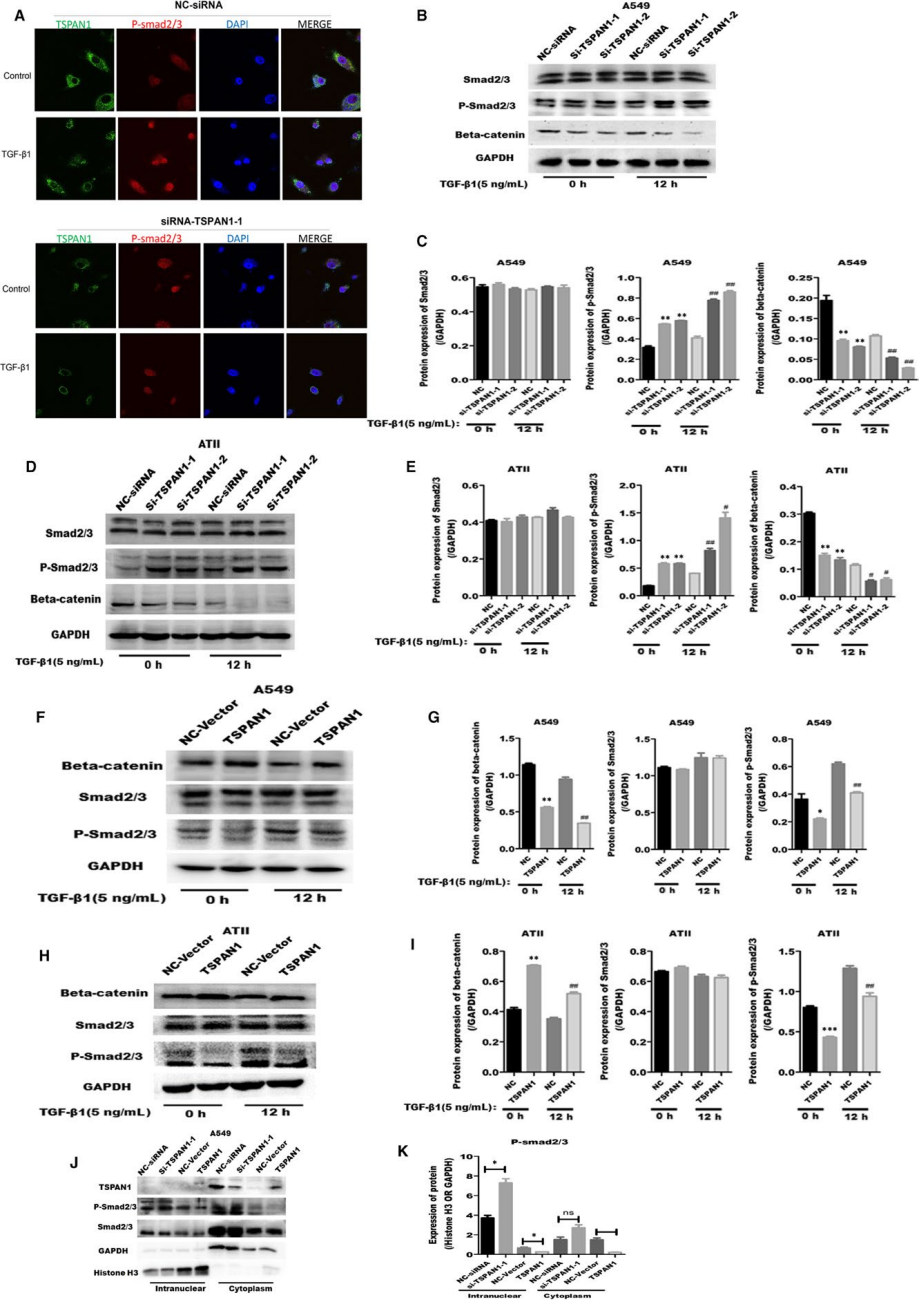
Tetraspanin 1 (TSPAN1) modulates beta‐catenin and phosphorylation of Smad2/3. Immunofluorescence staining analysis of TSPAN1 and phosphorylation of Smad2/3 after transfected with siRNA for TSPAN1 or negative control (NC) in A549 cells with or without TGF‐β_1_ treatment (5 ng/mL transforming growth factor‐β1 [TGF‐β_1_] or control) (green: TSPAN1; red: P‐smad2/3; blue: 4′,6‐diamidino‐2‐phenylindole). Magnification: 1200X. (B‐I) Western blot analysis of various proteins after transfected with siRNA or exogenous expression plasmid for TSPAN1 or NC in A549 and alveolar epithelial type II (ATII) cells with or without TGF‐β_1_ treatment (5 ng/mL; 0, 12, 24 h). B, Proteins of Smad2/3, beta‐catenin and phosphorylation of Smad2/3 were detected by silencing TSPAN1 in A549 cells. C, Quantification of the results in panel (A). D, The proteins expression of Smad2/3, beta‐catenin and phosphorylation of Smad2/3 were detected, using western blotting after silencing TSPAN1 in ATII cells. E, Quantification of the results in panel (D). F, Proteins of Smad2/3, beta‐catenin and phosphorylation of Smad2/3 were detected after overexpression of TSPAN1 in A549 cells. G, Quantification of the results in panel (F). H, Proteins of Smad2/3, beta‐catenin and phosphorylation of Smad2/3 were detected by Western blotting after overexpression of TSPAN1 in ATII cells. I, Quantification of the results in panel (H). J, Phosphorylation of Smad2/3 was detected by Western blotting in intranuclear or cytoplasm after silencing TSPAN1 in A549 cells. K, Quantification of the results in panel (J). The data are expressed as the means ± SEM (n = 3), with results representative of 3 independent experiments. (*P < 0.05, **P < 0.01, ***P < 0.001 vs NC 0 h; ^#^ *P < *0.05, ^##^ *P* < 0.01, ^###^ *P* < 0.001 vs NC 12 h)

Generally, activation of Wnt/beta‐catenin and TGF‐β/Smads pathway is the classical signal pathway in EMT. We also investigated relationship between TSPAN1 and Wnt/beta‐catenin or TGF‐β/Smads pathway. A549 and ATII cells were transfected with siRNA or exogenous‐expressed plasmid for TSPAN1 or NC. Firstly, we detected the expression of p‐smad2/3 and TSPAN1 by immunofluorescence after TSPAN1‐knockdown in A549 cells. It is observed that TGF‐β_1_ increased the expression of p‐smad2/3, promoted p‐smad2/3 into nuclear, and inhibited the expression of TSPAN1. Moreover, Knockdown TSPAN1 promoted exaltation of p‐smad2/3 induced by TGF‐β1 in A549 cells (Figure 5A). Then, we observed that the expression of Smad2/3 was not changed, but the phosphorylation of Smad2/3 was increased, the expression of beta‐catenin was decreased after silencing TSPAN1 compared to NC in A549 and ATII cells with or without TGF‐β_1_ for 12 hours (Figure 5B‐E). Moreover, after TSPAN1 was transfected in A549 and ATII cells, the expression of Smad2/3 was also unchanged, however, the phosphorylation of Smad2/3 and the degradation of beta‐catenin were inhibited under TGF‐β_1_ treatment (Figure 5F‐I). In addition, it was observed silencing TSPAN1 stimulated nuclear translocation of p‐smad2/3 (Figure 5J,K) and p‐smad2/3 was accumulated in nuclear in A549 cells. Taken together, TSPAN1‐mediated Wnt/beta‐catenin and TGF/Smads pathway by regulating the expression of beta‐catenin and phosphorylation of Smad2/3 and the exogenous expressed TSPAN1 suppressed TGF‐β_1_‐induced degradation of beta‐catenin and phosphorylation of Smad2/3 in A549 and ATII cells.

## 4 DISCUSSION

Idiopathic PF is a progressive lung disease characterized by fibroblast accumulation, collagen deposition and parenchyma destruction.^24^ Epithelial‐to‐mesenchymal transition has been implicated in the pathogenesis of fibrosis in various organs, including the lung.^25^ Although advances have been made in elucidating causes and mechanisms of EMT, potentially leading to some new treatment options,^9^, ^26^ however, the mechanism of EMT in IPF remain not entirely understood.

In our study, the GEO data (GSE32539) was used to analyse. We found TSPAN1 is an aberrant expression gene in human IPF tissue compared to normal lung tissue (Figure 1A,B), and we verified the lower protein expression of TSPAN1 in patient IPF tissue, bleomycin‐induced PF mouse and lung fibroblasts (Figure 1 E, H and K). This is the first time that TSPAN1 is implicated in the molecular pathogenesis of PF. Furthermore, we demonstrated that TSPAN1 inhibited TGF‐β_1_‐induced EMT characteristics by regulating Smad2/3 and beta‐catenin pathway in AECs.

Recently, down‐regulation of TSPAN1 expression markedly blocks GC cell proliferation, cell‐cycle progression and invasive activity in gastric cancer.^27^ However, it is observed opposite effect that TSPAN1 control cell migration in prostate cancer.^21^, ^28^ These reports confirmed that TSPAN1 participate cell migration and invasion in some human cancer, meantime, indicating TSPAN1 may be involved in complex signalling pathway and dependent‐tissue specificity. Epithelial‐to‐mesenchymal transition was considered to be a mechanism of epithelial cells' transformation in IPF. Therefore, we assumed that TSPAN1 may play the key role in EMT‐participated in the pathogenetics of PF.

Early reports proved EMT makes a potential contribution to collagen‐producing fibroblasts and myofibroblasts in IPF, and AECs can transdifferentiate into myofibroblasts when they acquire the mesenchymal phenotype via EMT.^29^, ^30^ In this study, we investigate whether the TSPAN1 mediate the transformation of AECs—a new possible mechanism for the pathogenetic of IPF, AECs transformed to fibroblast‐like cell, via the process of EMT in IPF. Injured epithelial cells prompt the fibrogenic process by releasing TGF‐β_1_, which is a prototypical profibrotic growth factor and well known for having a pivotal role in inducing EMT.^31^ We confirmed that reduced protein expression of TSPAN1 and increased protein of TGF‐β_1_ in mouse model of bleomycin‐induced PF and the lung tissue of patient with PF by immunohistochemically staining and Western blotting (Figure 1B,D‐G). Furthermore, we used A549 cells and primary rat ATII cells as the AECs model to evaluate the biology function of TSPAN1 in EMT. The protein expression of TSPAN1 is lower in fibroblasts, including MRC‐5 and rat primary lung fibroblast compared to A549 and ATII (Figure 1H,I), indicating the protein of teraspanin1 is less in fibroblasts. We observed that the gene and protein expressions of TSPAN1 were significantly reduced in A549 and ATII cells following TGF‐β_1_ treatment (Figure 1K,L). It implied that TSPAN1 may participate in TGF‐β_1_‐induced EMT in AECs. Previous studies indicated that after EMT mediating epithelial cells to become mesenchymal cells, the cells obtained migratory capability.^32^ Therefore, we also detected the change of cell migration in A549 cells by silencing or overexpressing TSPAN1. Knockdown‐TSPAN1 enhanced the wound healing and migratory capability in A549 cells without TGF‐β_1_ treatment (Figure 3A‐D), oppositely overexpressed‐TSPAN1 suppressed the wound healing and cell migratory capability induced by TGF‐β_1_ in A549 cells (Figure 4C‐F), indicating that TSPAN1 reduced the EMT potential of cells. Epithelial‐to‐mesenchymal transition is typically characterized by the loss of polarity, cytoskeleton reorganization, loss of epithelial morphology and markers, including E‐cadherin and zonula occludens‐1 and the gain of mesenchymal morphology and markers such as α‐SMA, vimentin and collagen I.^33^, ^34^ Previous reports indicated that increased expression of mesenchymal and reciprocal low expression of epithelial markers induces profound alteration in epithelial cell polarity and morphology, resulting into EMT.^32^ In this study, the protein expression of N‐cadherin, vimentin and α‐SMA was significantly up‐regulated by silencing TSPAN1 in A549 and ATII cells with or without TGF‐β_1_ treatment (Figure 2G,H,J). While overexpressed TSPAN1 could impede TGF‐β_1_‐induced the expression of α‐SMA in A549 cells and promoted the protein expression of E‐cadherin, we also observed that in the presence of exogenous TSPAN1, the protein expression of N‐cadherin and vimentin were reduced in A549 and ATII cells with or without TGF‐β_1_ treatment (Figure 3E‐H). Taken together, these results revealed a new function of TSPAN1, which regulate AECs transformation to the mesenchymal state. Moreover, we also observed that TSPAN1 could interfere in the process of TGF‐β_1_‐induced EMT in A549 and ATII cells (Figures 2E‐H & 3A‐H). It indicates that TSPAN1 may mediate TGF‐β_1_‐dependent signalling pathway to regulate EMT in AECs. TGF‐β signalling is a powerful inducer of EMT, mostly through its canonical Smad‐dependent pathway,^10^, ^11^ and activation of Wnt/beta‐catenin pathway also transmit extracellular and intracellular signals to mediate EMT in epithelial cells.^35^ Previous studies have indicated the TGF‐β_1_signals‐Smad2, β‐catenin signalling axis activate EMT and α‐SMA.^11^, ^36^ To explore the mechanism, the underlying TSPAN1‐mediated the EMT in lung epithelial cells, we further assumed that the action of TSPAN1 on EMT is involved in activating Smad2/3 and the beta‐catenin pathway, as shown in Figure 5, knockdown TSPAN1 promoted the phosphorylation of Smad2/3, but reduced the accumulation of beta‐catenin (Figure 5A,D), indicating the TM protein has a different action for profibrosis signalling pathways, even opposite action based on the TSPAN1 alone or combined with other proteins and the exogenous expression of TSPAN1‐blocked TGF‐β_1_‐induced the phosphorylation of Smad2/3 and the accumulation of beta‐catenin in A549 and ATII cells (Figure 5F‐I). These results showed that TSPAN1 modulated the TGF‐β/Smad2/3 and beta‐catenin signalling pathway in A549 and ATII cells. Some reports suggested that there are crosstalk and interaction between TGF‐β and β‐catenin signalling pathways in EMT, and TGF‐β_1_ activates β‐catenin‐dependent signalling, and that both pathways synergize to induce α‐SMA expression through direct binding of Smad3, beta‐catenin and CBP at the α‐SMA promoter.^13^, ^14^ Thence, we speculated that TSPAN1 may mediate the phosphorylation of Smad2/3 to impede interaction between Smad3 and beta‐catenin, following inhibition of EMT in AECs.

## 5 CONCLUSIONS

In conclusion, TSPAN1 may be an important signal conductor in pathogenesis of PF, and the TSPAN1 regulated EMT by impeding TGFβ_1_‐activated Smad2/3 and β‐catenin‐dependent signalling in AECs. However, we still have some uncertainty that whether the TSPAN1 suppress the binding of Smad3 and beta‐catenin and how the TSPAN1 regulates the interaction between Smad3 and beta‐catenin, more experimental data are required to answer these questions in future works. Based on these results, our study indicates that TSPAN1 may be a potential molecular therapy targets in PF.

## CONFLICT OF INTEREST

No conflicts of interest have been reported by the authors or by any individuals in control of the content of this article.

## AUTHOR CONTRIBUTION

Gang Liu, Yahong Wang, and Lawei Yang contributed equally to this study. All authors listed were involved in the study and preparation of the manuscript. All conference has been list at the end of the paper with mark in the manuscript. All data supporting the conclusions are included in this article. All procedures performed in studies involving animals were in accordance with the Ethics Committee of affiliated hospital, Guangdong Medical University, Zhanjiang, China.
